# Hypermethylation‐mediated down‐regulation of lncRNA TBX5‐AS1:2 in Tetralogy of Fallot inhibits cell proliferation by reducing *TBX5* expression

**DOI:** 10.1111/jcmm.15298

**Published:** 2020-05-05

**Authors:** Jing Ma, Shiyu Chen, Lili Hao, Wei Sheng, WeiCheng Chen, Xiaojing Ma, Bowen Zhang, Duan Ma, Guoying Huang

**Affiliations:** ^1^ Department of Facial Plastic and Reconstructive Surgery ENT Institute Eye & ENT Hospital Fudan University Shanghai China; ^2^ Department of Biochemistry and Molecular Biology Research Center for Birth Defects Institutes of Biomedical Sciences Key Laboratory of Metabolism and Molecular Medicine Ministry of Education School of Basic Medical Sciences Fudan University Shanghai China; ^3^ Children’s Hospital of Fudan University Shanghai China

**Keywords:** cell proliferation, lncRNA TBX5‐AS1:2, RNA‐RNA interaction, *TBX5*, TOF

## Abstract

Tetralogy of Fallot (TOF) is the most common complex congenital heart disease (CHD) with uncertain cause. Although long non‐coding RNAs (lncRNAs) have been implicated in heart development and several CHDs, their role in TOF is not well understood. This study aimed to investigate how dysregulated lncRNAs contribute to TOF. Using Gene Expression Omnibus data mining, bioinformatics analysis and clinical heart tissue sample detecting, we identified a novel antisense lncRNA TBX5‐AS1:2 with unknown function that was significantly down‐regulated in injured cardiac tissues from TOF patients. LncRNA TBX5‐AS1:2 was mainly located in the nucleus of the human embryonic kidney 293 (HEK293T) cells and formed an RNA‐RNA double‐stranded structure in the overlapping region with its sense mRNA T‐box transcription factor 5 (*TBX5*), which is an important regulator in heart development. Knock‐down of lncRNA TBX5‐AS1:2 via promoter hypermethylation reduced *TBX5* expression at both the mRNA and protein levels by affecting its mRNA stability through RNA‐RNA interaction. Moreover, lncRNA TBX5‐AS1:2 knock‐down inhibited the proliferation of HEK293T cells. In conclusion, these results indicated that lncRNA TBX5‐AS1:2 may be involved in TOF by affecting cell proliferation by targeting *TBX5*.

## 1 INTRODUCTION

Congenital heart disease (CHD) is one of the commonest human congenital anomalies, with a morbidity of six to eight per 1000 live births, and nearly, a third of all major congenital malformations are accompanied by cardiac abnormalities.^1^ CHD comprises a group of structural heart and great vessel disorders caused by cardiovascular dysplasia during the embryonic period.

Tetralogy of Fallot (TOF) is the most frequent complex CHD accounting for 7%‐10% of all CHDs,^2^, ^3^ with an estimated incidence among live births of three per 10,000.^3^ TOF was one of the first‐described CHDs and was named after the French physician Dr Etienne‐Louis Arthur Fallot,^4^, ^5^ who characterized the four typical anatomical features of TOF: (a) ventricular septal defect; (b) biventricular connection (overriding) of the aorta; (c) right ventricular outflow tract obstruction; and (d) right ventricular hypertrophy.^6^ TOF was also one of the first CHDs to be successfully repaired by surgery.^5^ Although the post‐operative survival rate among TOF patients has been greatly improved due to advances in surgical techniques, the incidence of late cardiac death in long‐term survivors continues to increase.^7^ There is thus an urgent need to explore the aetiology and pathogenesis of TOF. The precise cause of TOF is currently unclear. As a multifactorial disease involving genetic‐environmental interactions,^8^ TOF may be related to chromosome aneuploidy (eg trisomy of chromosomes 21, 18, or 13) or to mutations of several genes (eg *NKX2.5*, *GATA4*, *TBX5*, *HAND2, JAG1, NOTCH and VEGF*) encoding transcription factors or components of signalling pathways.^9^, ^10^, ^11^, ^12^ However, single‐gene mutations occur in only a small minority of TOF patients, and more complex dysregulation of multiple genes is more common. Epigenetic modification plays an important role in gene expression and provides a bridge and mechanism for genetic and environmental interactions. There are three main types of epigenetic modifications: DNA methylation, histone modification and non‐coding RNAs. However, the role of epigenetic factors in CHDs (including TOF) remains largely unclear. Abnormal gene expression during cardiac development leading to CHD may due to changes in the epigenetic landscape surrounding the genes’ regulatory regions,^2^ and more complex causes of TOF may therefore be associated with epigenetic variation. A few studies have investigated the role of epigenetic modifications in TOF.^1^, ^13^, ^14^, ^15^, ^16^ We previously found that methylation abnormalities in multiple genes were involved in the pathogenesis of TOF^9^, ^17^, ^18^, ^19^ and detected abnormal expression of some microRNAs (miRNAs) and significant changes in histone modification in injured heart tissues from TOF patients.^20^, ^21^, ^22^ As the largest class and most important component of non‐coding RNAs, long non‐coding RNAs (lncRNAs) of over 200 nucleotides are numerous in eukaryotes and function as transcriptional regulators in many cell processes and diseases.^23^ Dysregulation of numerous lncRNAs has been shown to participate in mammalian cardiogenesis and in the pathogenesis of related diseases. Differential expression of lncRNAs in heart tissue of CHD can regulate gene expression in several ways. Enhancer lncRNAs specifically regulate chromatin state transition during cardiac development, and participate in the differentiation of embryonic stem cells into heart muscle and in cardiac remodelling, whereas decoy lncRNAs, guide lncRNAs and scaffold lncRNAs affect the activity of cardiac transcription factors by binding protein factors. LncRNAs can also modulate cardiac development via the lncRNA‐miRNA‐mRNA co‐expression network.^24^ However, there has been only one previous report on the role of lncRNAs in TOF,^16^ and their function in TOF therefore remains largely unknown.

In the current study, we characterized the lncRNA and mRNA profiles in human foetal and adult heart tissues by Gene Expression Omnibus (GEO) data mining and bioinformatics analysis, and focused on a previously unreported antisense lncRNA TBX5‐AS1:2 with unknown function. LncRNA TBX5‐AS1:2 was significantly up‐regulated in foetal heart and was predicted to adjust the expression of its sense gene *TBX5*, which is one of the vital transcription factors related to cardiac development. We further demonstrated that lncRNA TBX5‐AS1:2 expression was markedly decreased in injured heart tissue from patients with TOF. In vitro, lncRNA TBX5‐AS1:2 down‐regulation, mediated by DNA hypermethylation in the promoter region, significantly suppressed cell proliferation by reducing the expression of *TBX5* at both the mRNA and protein levels, the reason of which was that lncRNA TBX5‐AS1:2 affected the stability of *TBX5* mRNA through the formation of an RNA‐RNA duplex.

## 2 MATERIALS AND METHODS

### 2.1 Data mining in GEO database and bioinformatics analysis

Online data mining was performed in the GEO database (https://www.ncbi.nlm.nih.gov/geo/) using the keywords lncRNA, human, heart development or CHD. Differentially expressed lncRNAs were analysed using the DESeq package, and WikiPathways database was applied to screen mRNAs related to heart development or CHD with differential expression. A coding and non‐coding co‐expression (CNC) network was established followed by these procedures: (a) data pre‐processing: for same gene, median value of different transcripts for same genes represents gene expression value; (b) data screening: assessing differential expression of lncRNA and mRNA; (c) calculation and removal of subset of data based on Pearson's correlation coefficient (PCC) and calculation of correlation coefficient of PCC between lncRNA coding genes using R values; (d) screening with a standard of PCC ≥0.9 or ≤−0.9 and *P* ≤ .05 as meaningful subset and constructing CNC network using Cytoscape. Meanwhile, mRNAs adjacent to lncRNAs in the CNC network (≤10 kbp on the genome) were annotated. Target lncRNA was selected based on the following requirements: (a) in the CNC network; (b) adjacent mRNA was one of the differentially expressed in relation to heart development or CHD; and (c) adjacent mRNA co‐expressed with the lncRNA.

### 2.2 Study subjects and samples

This study was reviewed and approved by the Institutional Research Ethics Committee of the Children's Hospital of Fudan University (2016‐56). Cardiac tissue samples were obtained from 53 patients with TOF from the Biobank of the Children's Hospital of Fudan University, Shanghai, China. The diagnosis of TOF was based on echocardiography carried out at our hospital. None of the patients included in the study had been diagnosed with extracardiac anomalies or had any common chromosomal anomalies, such as 22q11 microdeletion. Cardiac tissues were removed from the blocked right ventricular outflow tract during surgery. Thirteen normal cardiac tissues samples were acquired from the Department of Forensic Medicine, Fudan University, Shanghai, China. They died as a result of traffic accidents and had no abnormal cardiac structures at autopsy. All tissue samples for RNA extraction were maintained in RNAlater^®^ RNA Stabilization Solution (Thermo Fisher Scientific) after surgery or autopsy and were stored at −80°C.

### 2.3 RNA extraction and quantitative polymerase chain reaction

Total RNA was extracted from cardiac tissues and cells using TRIzol reagent (Invitrogen) according to the manufacturer's instructions. The quantity and quality of the extracted RNA were assayed using a NanoDrop ND‐2000 spectrophotometer (Thermo Fisher Scientific) and agarose gel electrophoresis. A total of 1000 ng of RNA from each sample was used to synthesize cDNA using a Prime Script RT Reagent Kit (Takara), followed by quantitative polymerase chain reaction (qPCR) with SYBR Premix Ex Taq™ (Takara) on a StepOnePlus™ Real‐Time PCR System (Thermo Fisher Scientific). Gene expression was normalized to the housekeeping gene glyceraldehyde 3‐phosphate dehydrogenase (*GAPDH*) and analysed according to the relative quantification method (2^−ΔΔCt^). The 2^−ΔΔCt^ was used to calculate the relative expression of RNA. Three repeated measurements were carried out for each sample.

The primers used were as follows:

lncRNA TBX5‐AS1:2‐F: GCGCCGAGAGAAGAGCTAGG,

lncRNA TBX5‐AS1:2‐R: CCTCGGCTCAGAGGTCAAGT;

TBX5‐F: AAAAGACCTGCCCTGCGATT,

TBX5‐R: TTTGATTCCCTCCATGCCCT;

GAPDH‐F: GGGAGCCAAAAGGGTCAT,

GAPDH‐R: GAGTCCTTCCACGATACCAA;

U1‐F: GACGGGAAAAGATTGAGCGG,

U1‐R: GCCACGAAGAGAGTCTTGAAGG;

Actin‐F: CATGTACGTTGCTATCCAGGC,

Actin‐R: CTCCTTAATGTCACGCACGAT.

### 2.4 Cell culture

Human embryonic kidney 293 (HEK293T) cells were cultured in a mixture of Dulbecco's modified Eagle's medium (Biological Industries, Kibbutz Beit Haemek), 10% foetal bovine serum (Biological Industries) and 1% penicillin‐streptomycin (Biological Industries) at 37°C in 5% CO_2_. All cell culture dishes and culture plates were purchased from Hangzhou Xinyou Biotechnology Co., Ltd.

### 2.5 Construction of eukaryotic overexpression vector and transient transfection

Human lncRNA TBX5‐AS1:2‐pcDNA 3.1 and human TBX5‐pcDNA 3.1 were obtained from GeneRay. The plasmids were sequenced and shown to be consistent with the sequence in the National Center for Biotechnology Information database (https://www.ncbi.nlm.nih.gov/). Empty vector (pcDNA 3.1) was used as a negative control (NC). When the cells plated in 6‐cm dishes reached about 75% confluence, they were transfected with plasmids using Lipofectamine 3000 (Invitrogen). The cells were harvested 48 hours after transfection.

### 2.6 Construction of lentiviral interference vector and stable cell line

Three short hairpin RNAs (shRNAs) targeting lncRNA TBX5‐AS1:2 (excluding the overlapping regions with *TBX5*) were designed and cloned into the lentiviral vector PGMLV‐SC5‐GFP by Genomeditech. The lentivirus was amplified in HEK293T cells and concentrated using polyethylene glycol (System Biosciences). The interference efficiency was detected by qPCR. HEK293T cells infected by the most interference‐efficient lentivirus were used to establish stable cell lines by selection with puromycin (Sigma‐Aldrich).

The lncRNA TBX5‐AS1:2 shRNA sequences used were as follows:

lncRNA TBX5‐AS1:2‐shRNA1: GGTGAGACATTCCCTGGTTTC,

lncRNA TBX5‐AS1:2‐shRNA2: GGAACACAGTATGTCTCTTCC,

lncRNA TBX5‐AS1:2‐shRNA3: GCTCTCCTCATTCATGTTAGT.

### 2.7 Cell proliferation assays

Cell proliferation was determined using a Cell Counting Kit‐8 (CCK8; Dojindo) according to the manufacturer's protocol. A total of 1 × 10^4^ HEK293T cells per well were seeded into 96‐well plates for adherence. CCK8 reagent (10 µL) was added to each well followed by incubation for 3 hours at 37°C. Cell viability was used to represent for proliferation and evaluated by the absorbance at 450 nm. All samples were prepared in triplicate and normalized to blank controls. Three time‐points were set at 40, 56 and 72 hours after transfection or incubation. The experiments were repeated three times.

### 2.8 Cell apoptosis assays

Cell apoptosis was detected by harvesting 1 × 10^6^ HEK293T cells and staining using an Annexin V‐fluorescein isothiocyanate (FITC)/propidium iodide (PI) Kit (Dojindo). Cells were stained successively with 5 µL FITC reagent and 5 µL PI reagent, and incubated in the dark for 15 minutes at room temperature each time. Flow cytometry analysis was performed using a FACSCalibur (BD Biosciences) and apoptosis was analysed using FlowJo software.

### 2.9 Nuclear‐cytoplasmic separation

Nuclear and cytoplasmic fractions were isolated from HEK293T cells using a PARIS kit (Thermo Fisher Scientific) according to the manufacturer's instructions. Actin was used as a cytoplasmic control, and U1 was used as a nuclear control. RNA levels of lncRNA TBX5‐AS1:2, Actin and U1 in the cytoplasm and nuclear components were measured by qPCR after RNA extraction and reverse transcription.

### 2.10 RNA fluorescence in situ hybridization (FISH) assay

Cells were grown in a 4‐chamber slides for 24 hours, fixed with 4% paraformaldehyde for 20 minutes and dehydrated in an ascending series of ethanol solutions. Cells were hybridized overnight at 42°C with probe. Non‐specific probe was removed using 0.5× saline sodium citrate containing 50% formamide at 37°C. Biotin‐labelled lncRNA TBX5‐AS1:2 was detected using anti‐biotin monoclonal antibody and secondary antibody. Finally, the slides were stained with DAPI (Cell Signaling Technology) and subjected to fluorescence signal detection under Leica TCS SP8 laser confocal microscope (Leica).The probe of lncRNA TBX5‐AS1:2 used was as follow:

CY3‐GGUUUCGAUUAAGAUACACCAUAGGCUCUACACGAUCGGC.

### 2.11 Western blot and antibodies

Human embryonic kidney 293 cells were collected and lysed with RIPA buffer (Yeasen) containing a protease inhibitor cocktail (Sigma‐Aldrich). Proteins were determined using a BCA Protein Assay Kit (Pierce) and equivalent amounts of proteins were separated by 10% sodium dodecyl sulphate‐polyacrylamide gel electrophoresis and transferred to nitrocellulose membranes (Pall). The membranes were blocked with 5% milk in Tris‐buffered saline Tween for 1 hour at room temperature and then incubated with specific primary antibodies at 4°C overnight, followed by secondary antibodies at room temperature for 1 hour. The primary specific antibodies used were anti‐TBX5 (1:1000, Novus) and anti‐Actin (1:8000, Proteintech), and the secondary horseradish peroxidase‐conjugated antibody was antimouse IgG (1:5000, Kangwei). The proteins were then visualized on X‐ray film using Clinx ChemiScope (Clinx Science Instruments).

### 2.12 Ribonuclease protection assay

Each RNA sample from HEK293T cells was incubated for 1 hour at 37°C and then treated with RNAse A+T cocktail (Ambion) to digest single‐stranded but not duplex RNAs. After incubation for 30 minutes at 37°C, samples were dealt with proteinase K (Yeasen). A NC without RNAse treatment was operated in the same way. After ribonuclease protection assay (RPA), reverse transcription (RT)‐PCR and gel electrophoresis were used to detect *TBX5* employing two sets of distinct primers designed to target the overlapping region of the *TBX5* sense and antisense transcripts, and the non‐overlapping region of *TBX5*, respectively. The primer sequences were as follows:

TBX5‐overlapping region‐F: CCCTCATTCCTCCGGAGAAAG,

TBX5‐overlapping region‐R: GCCGGGTCTGCGCAGCCACAG;

TBX5‐non‐overlapping region‐F: ACATCGTGAAAGCGGATGAA,

TBX5‐non‐overlapping region‐R: GTGATCTTGTGGTTCTGGTAGG.

### 2.13 RNA‐RNA pull‐down assay

Full‐length biotinylated lncRNAs and antisense transcripts were transcribed using an AmpliScribe™ T7‐Flash™ Biotin‐RNA Transcription Kit (Epicentre Technologies). After purification, HEK293T cells were lysed for 40 minutes with RNA pull‐down buffer (20 mmol/L Tris pH7.5, 100 mmol/L KCl, 5 mmol/L MgCl_2_, 0.5% NP‐40, RNase Inhibitor 160 U/mL cocktail). The supernatant was mixed with biotinylated lncRNAs and antisense transcripts for 2 hours at 4°C, followed by the addition of Dynabeads MyOne Streptavidin T1 (Invitrogen) and incubation overnight at 4°C. After washing beads four times, RNA was extracted using TRIzol reagent. The lncRNA‐associated RNAs were subjected to cDNA synthesis and qPCR using the following primers:

sense lncRNA TBX5‐AS1:2‐F:

TAATACGACTCACTATAGGGGCCGGGTCTGCGCAGCCA,

sense lncRNA TBX5‐AS1:2‐R:TGCAATCAGAAATATTAT;

antisense lncRNA TBX5‐AS1:2‐F:

TAATACGACTCACTATAGGGTGCAATCAGAAATATTAT,

antisense lncRNA TBX5‐AS1:2‐R:GCCGGGTCTGCGCAGCCA.

### 2.14 Measurement of RNA stability

RNA stability was measured by plating HEK293T cells at 2.5 × 10^5^ cells per well in 6‐well plates and culturing overnight, followed by incubation with actinomycin D (Sigma‐Aldrich) for 4, 6, 8 and 10 hours, respectively. Total RNA was extracted after treatment at each time‐point and subjected to qPCR for *TBX5* mRNA quantification.

### 2.15 Analysis of lncRNA TBX5‐AS1:2 promoter methylation status

The DNA sequence of the lncRNA TBX5‐AS1:2 regulatory region was obtained from the GenBank database (https://www.ncbi.nlm.nih.gov/nuccore/NC_000012.12?from=114408195&to=114412832&report=genbank), and CpG islands were predicted using MethPrimer (http://www.urogene.org/cgibin/methprimer/methprimer.cgi). The DNA methylation statuses of the selected CpG islands were analysed by bisulphite sequencing PCR (BSP). Genomic DNA extracted from six TOF and five normal cardiac tissues was subjected to bisulphite conversion using an EpiTect Fast DNA Bisulfite Kit (Qiagen) according to the manufacturer's instructions. LncRNA TBX5‐AS1:2 CpG islands from bisulphite‐modified DNA were then amplified by EpiTaq HS DNA polymerase (Takara), and the PCR products were purified and cloned into the T/A cloning vector pGEM T‐Easy (Promega). Ten positive clones were isolated and sequenced. Methylation analysis was performed using BiQ Analyzer 2.0 and QUMA (http://quma.cdb.riken.jp/). The primers used were as follows: lncRNA TBX5‐AS1:2‐I2‐F1: TTTTAGTAAAATAAAGAGGTAATTAGG,

lncRNA TBX5‐AS1:2‐I2‐R1: AAAATCTAAAATAAACTCCCACCTC;

lncRNA TBX5‐AS1:2‐I3‐F1: GAGGAGTTTTGGGTAAATGAATAT,

lncRNA TBX5‐AS1:2‐I3‐R1: AATTACAAAACAAAATAAAATACCTC.

### 2.16 Construction of dual‐luciferase reporter plasmids

A lncRNA TBX5‐AS1:2 DNA fragment containing CpG island 2 was amplified and cloned into the pGL3‐Basic‐firefly vector (Promega). The recombinant plasmid was treated with CpG methyltransferase (M.SssI) (New England Biolabs) for 2 hours at 37°C and then purified using an AxyPrep DNA Gel Extraction Kit (Axygen) to generate a patch‐methylated construct. Whether the plasmids were methylated was detected by methylation‐sensitive restriction enzymes (MSREs) (Xho I and Sal I) (New England Biolabs) digestion and DNA gel electrophoresis assay. Methylation efficiency was evaluated by BSP and the PCR products were used for direct pyrosequencing. The primers used were as follows: lncRNA TBX5‐AS1:2‐I2‐F2‐1: TGTGAATYGATAGTATTAATATAYGTTT,

lncRNA TBX5‐AS1:2‐I2‐R2‐1‐tail:

aaccttcaacaccccaaccatataTAATTTATATCTTTATTTATTCCCRAAACC;

lncRNA TBX5‐AS1:2‐I2‐F2‐2: TTAGTGTAAGTGTAGGTGTTAGAATATT,

lncRNA TBX5‐AS1:2‐I2‐R2‐2‐biotin: aaccttcaacaccccaaccatata.

(Y = C or T, R = A or G).

### 2.17 Dual‐luciferase reporter assay

Human embryonic kidney 293 cells at 1 × 10^4^ cells per well were seeded in 96‐well plates and incubated at 37°C overnight. The respective methylated and unmethylated reporter plasmids were co‐transfected with pGL3‐Renilla vector into HEK293T cells using Lipofectamine 3000. After transfection for 48 hours, the HEK293T cells were treated with a Dual‐Luciferase Reporter Assay System (Promega) according to the manufacturer's protocol. Both firefly and Renilla luciferase activities were measured using an EnSpire plate reader (PerkinElmer). All samples were prepared in triplicate. The firefly luciferase activity normalized to the Renilla luciferase activity represented the transcriptional activity of the lncRNA TBX5‐AS1:2.

### 2.18 Statistical analysis

All experiments were repeated three times. All statistical analyses were performed using paired two‐tailed Student's *t* tests with GraphPad Software. Data were shown as mean ± standard error (SEM), and a *P* value < .05 was considered statistically significant.

## 3 RESULTS

### 3.1 LncRNA TBX5‐AS1:2 was selected by data mining and bioinformatics analysis

**FIGURE 1:**
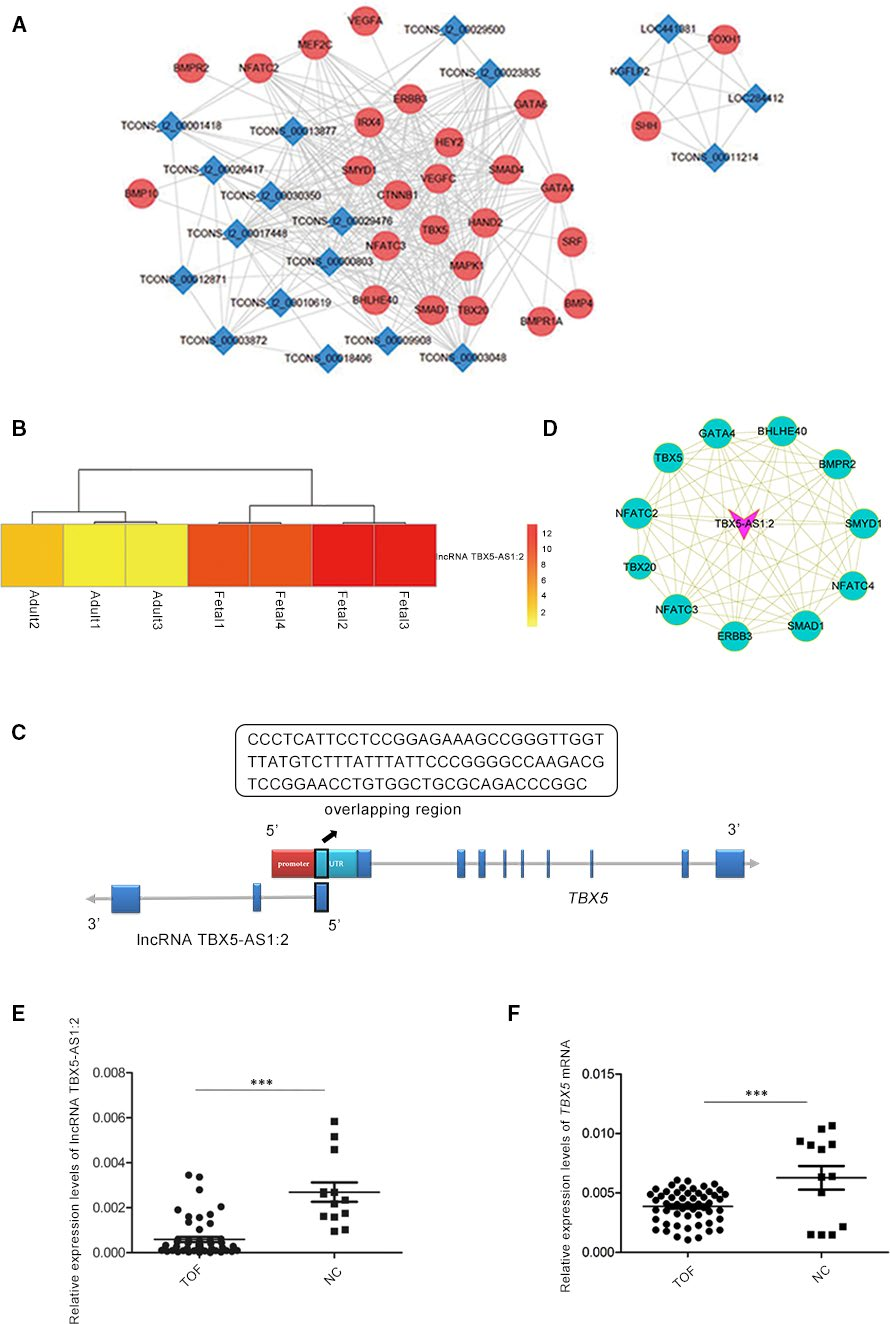
LncRNA TBX5‐AS1:2 was selected by data mining of the GEO database and bioinformatics analysis. A, CNC network of 19 lncRNAs and 26 mRNAs with a standard of PCC ≥0.9 or ≤−0.9 and *P* ≤ .05. Blue rhombus represents lncRNAs, red circular nodes represent mRNAs, and lines indicate gene co‐expression relationship between lncRNA and mRNA. B, Higher expression of lncRNA TBX5‐AS1:2 in foetal compared with adult hearts. Expression values represented by red and yellow shades, indicating expression above and below the median across all samples, respectively. C, Relative positions of lncRNA TBX5‐AS1:2 and its nearby gene *TBX5* on the chromosome. LncRNA TBX5‐AS1:2 is localized at the antisense chain of the coding gene *TBX5*, with overlapping and complementary regions. Black highlighted region indicates the overlapping region (92 bp), and its sequence is presented in the above box. Blue boxes indicate exons. D, CNC network of lncRNA TBX5‐AS1:2 and 11 mRNAs. Rose V represents lncRNA TBX5‐AS1:2, green circular nodes represent mRNAs, and node size indicates the gene expression level (larger dot, higher expression level). Lines represent the gene co‐expression relationship between lncRNA TBX5‐AS1:2 and mRNA (full lines, positive correlation; dashed lines, negative correlation). E and F, LncRNA TBX5‐AS1:2 and *TBX5* were significantly down‐regulated in TOF cardiac tissue samples compared with normal control (NC) by qPCR analysis. Values are mean ± SEM, n = 3, ***P < .0001. CNC, coding and non‐coding; GEO, Gene Expression Omnibus; lncRNAs, long non‐coding RNAs; PCC, Pearson's correlation coefficient; qPCR, quantitative polymerase chain reaction; TOF, Tetralogy of Fallot

To gain an insight into the role of lncRNAs in heart development or CHD, we mined data in the GEO database and a result of transcriptome sequencing of two human foetal hearts (https://www.ncbi.nlm.nih.gov/geo/query/acc.cgi?acc=GSE68279) was filtered out, which was from an article related to the lncRNA profile in human foetal and adult hearts.^25^ Two other human foetal heart RNA‐Seq data sets (GSM1059494, 17 weeks and GSM1059495, 13 weeks) and three normal adult heart RNA‐Seq datasets (GSM1101970, GSM1698563, and GSM1698564) were also analysed in this article. Further bioinformatics analysis of the RNA‐Seq data for the seven samples identified 277 lncRNAs and 47 mRNAs related to heart development or CHD that were differentially expressed between foetal and adult heart tissues (Table S1 and S2). The CNC network including 19 lncRNAs and 26 mRNAs that met the criteria suggested that lncRNAs and mRNAs may have regulatory relationships with important roles in cardiogenesis or CHD (Figure 1A). Among the 19 lncRNAs, lncRNA TBX5‐AS1:2 was highly expressed in foetal heart tissues (Figure 1B) and was selected for further analysis based on the inclusion requirements we set. Its neighbour gene was *TBX5* (Figure 1C), which encodes a transcription factor involved in the control of cardiogenesis and related to CHD. LncRNA TBX5‐AS1:2 was transcribed from the negative strand of the sense gene *TBX5* in a head‐to‐head orientation. Their overlapping region was 92 bp long and covered exon1 of lncRNA TBX5‐AS1:2 and part of the 5′ untranslated region of *TBX5* (Figure 1C). The CNC network of lncRNA TBX5‐AS1:2 indicated that it may also act on *TBX5* (Figure 1D).

### 3.2 LncRNA TBX5‐AS1:2 and *TBX5* were down‐regulated in TOF heart tissues

To validate the role of lncRNA TBX5‐AS1:2 in heart development or TOF and to determine whether lncRNA TBX5‐AS1:2 affected the expression of *TBX5* in TOF, we detected lncRNA TBX5‐AS1:2 and *TBX5* expression in 53 samples of injured heart tissue from patients with TOF and 13 normal heart samples using qPCR. The results confirmed that lncRNA TBX5‐AS1:2 and *TBX5* expression were significantly down‐regulated in TOF compared with normal control (NC) (Figure 1E,F).

### 3.3 LncRNA TBX5‐AS1:2 knock‐down inhibited cell proliferation in vitro

**FIGURE 2:**
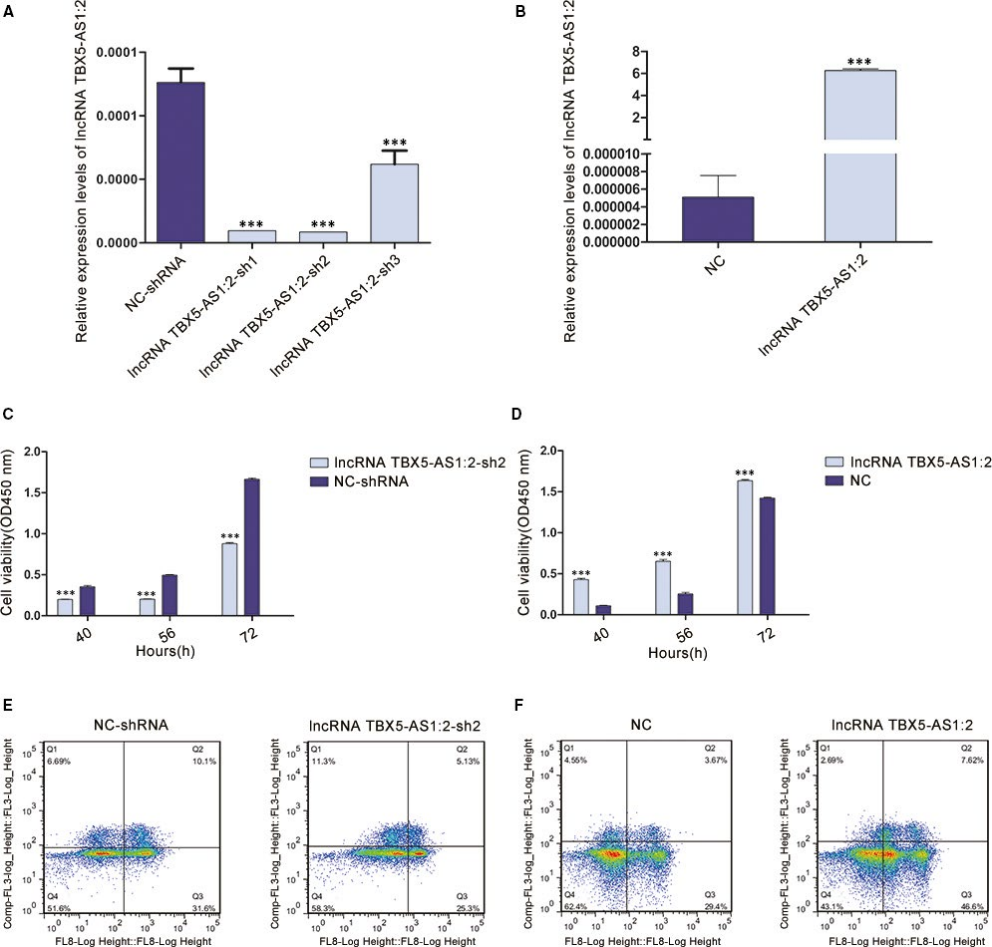
LncRNA TBX5‐AS1:2 affected proliferation of HEK293T cells. A, Three shRNAs targeting the non‐overlapping regions of lncRNA TBX5‐AS1:2 were designed to knock‐down lncRNA TBX5‐AS1:2 expression. The shRNA2 with the greatest interference efficiency was used to establish stable cell lines. B, LncRNA TBX5‐AS1:2 was successfully overexpressed. C and D Positive regulation of cell proliferation by lncRNA TBX5‐AS1:2 was revealed by CCK8 assays. E and F, LncRNA TBX5‐AS1:2 had no effect on cell apoptosis according to flow cytometry analysis. Values are mean ± SEM, n = 3, ***P < .0001. HEK293T, human embryonic kidney 293; lncRNAs, long non‐coding RNAs; shRNAs, short hairpin RNAs

Abnormal cell proliferation and apoptosis is a key feature of TOF. To investigate the effects of lncRNA TBX5‐AS1:2 on cell proliferation and apoptosis, lncRNA TBX5‐AS1:2 was successfully down‐regulated by stable transfection of shRNA2 lentivirus with the most interference efficiency and up‐regulated separately in HEK293T cells (Figure 2A,B). CCK8 assay showed that lncRNA TBX5‐AS1:2 knock‐down significantly inhibited cell proliferation, consistent with the tendency in cells overexpressing lncRNA TBX5‐AS1:2 (Figure 2C,D). However, flow cytometry analysis revealed that lncRNA TBX5‐AS1:2 dysregulation did not affect apoptosis of HEK293T cells (Figure 2E,F).

### 3.4 LncRNA TBX5‐AS1:2 was mainly located in the nucleus of HEK293T cells

**FIGURE 3:**
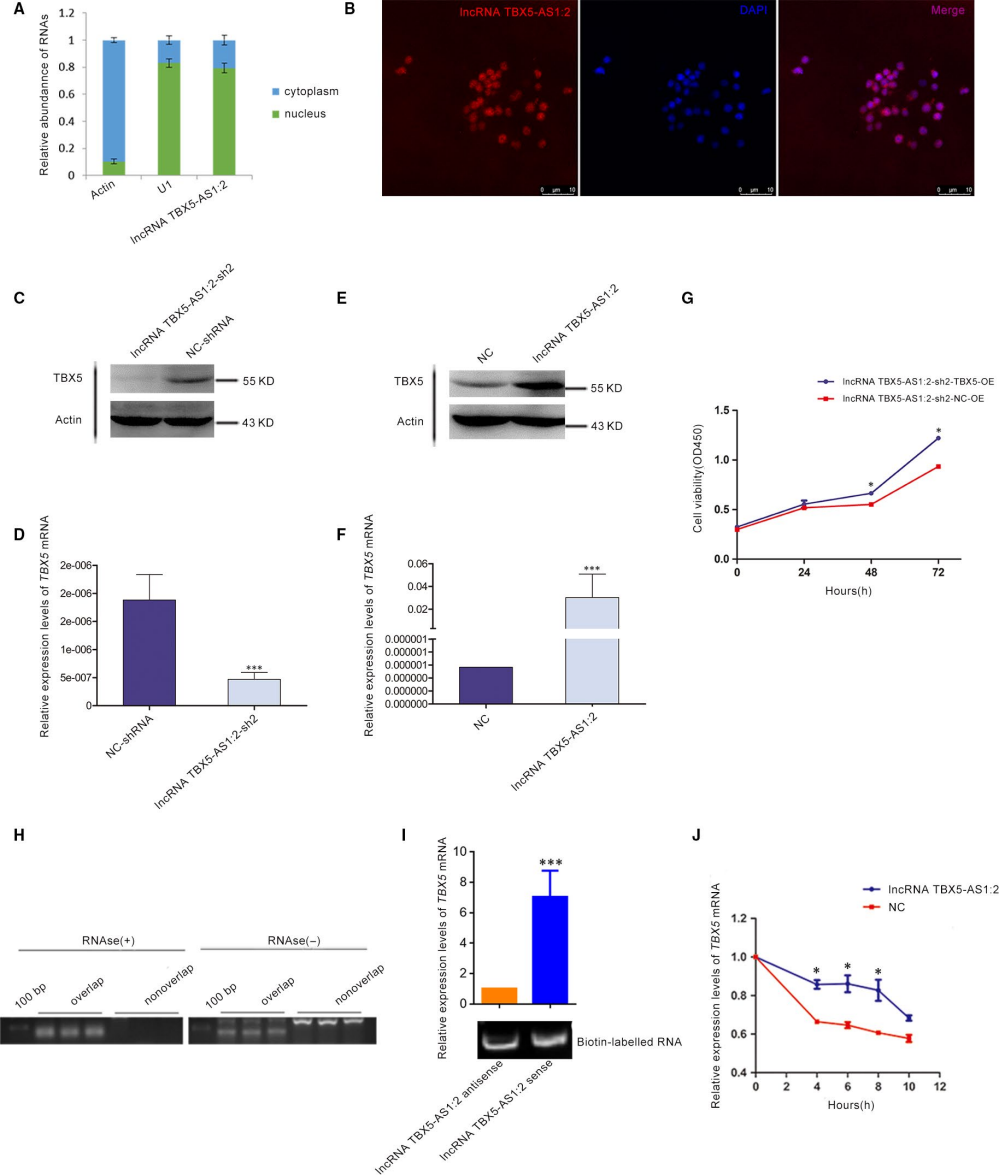
LncRNA TBX5‐AS1:2 regulated expression of *TBX5* in mRNA and protein levels by forming an RNA duplex with *TBX5* to increase its stability. A, Nucleus cytoplasm separation indicated that lncRNA TBX5‐AS1:2 was mainly located in the nucleus of HEK293T cells, similar to U1. B, The result of a RNA‐FISH assay also showed that lncRNA TBX5‐AS1:2 was almost nuclear in HEK293T cells. Centre DAPI was used to stain nuclei (blue); left red fluorescence was from the biotin fusions; right the merged image. C and E, Dysregulation of lncRNA TBX5‐AS1:2 positively regulated TBX5 protein levels according to WB results. D and F, Dysregulation of lncRNA TBX5‐AS1:2 positively regulated *TBX5* mRNA levels by qPCR analysis. Values are mean ± SEM, n = 3, ***P < .0001. G, Reduced cell proliferation caused by down‐regulation of lncRNA TBX5‐AS1:2 was rescued by *TBX5* overexpression in HEK293T cells. H, RPA and RT‐PCR revealed that the overlapping region of lncRNA TBX5‐AS1:2 and *TBX5* mRNA could not be digested by RNAse, suggesting the formation of an RNA‐RNA duplex. RNAse(+) indicates RNAse treatment; RNAse(‐) indicates no RNAse treatment. I, RNA‐RNA pull‐down assay showed the in vitro interaction between lncRNA TBX5‐AS1:2 and *TBX5*. J, LncRNA TBX5‐AS1:2 increased the stability of *TBX5* mRNA in HEK293T cells during 10 h after blocking new RNA synthesis with actinomycin D. HEK293T, human embryonic kidney 293; lncRNAs, long non‐coding RNAs; qPCR, quantitative polymerase chain reaction; RPA, ribonuclease protection assay; WB, Western blotRNA‐FISH

To further explore the underlying mechanism of lncRNA TBX5‐AS1:2 affecting cell proliferation involved in TOF, subcellular localization of lncRNA TBX5‐AS1:2, which determines its action mode, was confirmed firstly. Nucleus cytoplasm separation indicated that lncRNA TBX5‐AS1:2 was mainly distributed in the nucleus in HEK293T cells (Figure 3A), and this was verified by RNA‐FISH assay (Figure 3B).

### 3.5 LncRNA TBX5‐AS1:2 knock‐down reduced TBX5 mRNA and protein levels

Previous bioinformatics prediction indicated that lncRNA TBX5‐AS1:2 may be co‐expressed with its sense gene *TBX5*. We therefore detected TBX5 mRNA and protein expression levels by qPCR and Western blot (WB) in HEK293T cells with down‐regulated and up‐regulated lncRNA TBX5‐AS1:2. *TBX5* mRNA and protein levels were both significantly down‐regulated after lncRNA TBX5‐AS1:2 knock‐down (Figure 3C,D) and up‐regulated after lncRNA TBX5‐AS1:2 overexpression (Figure 3E,F).

### 3.6 LncRNA TBX5‐AS1:2 affecting cell proliferation was mediated by *TBX5*

Considering that lncRNA TBX5‐AS1:2 regulated *TBX5* expression, we performed *TBX5* rescue experiment to determine whether lncRNA TBX5‐AS1:2 influencing cell proliferation was mediated by *TBX5. TBX5* was overexpressed successfully in HEK293T cells with lncRNA TBX5‐AS1:2 knock‐down, and then, CCK8 assay showed that *TBX5* rescued the cell proliferation suppressed by lncRNA TBX5‐AS1:2 knock‐down (Figure 3G). Therefore, it indicated that lncRNA TBX5‐AS1:2 knoc‐kdown may inhibit cell proliferation by *TBX5*.

### 3.7 LncRNA TBX5‐AS1:2 influenced *TBX5* mRNA stability by RNA duplex formation

Antisense lncRNA can hybridize with its sense mRNA to form an RNA duplex that protects the mRNA from RNase degradation. LncRNA can influence mRNA stability via this RNA‐RNA interaction to modulate sense mRNA expression. We therefore verified the formation of a protective lncRNA TBX5‐AS1:2 and *TBX5* mRNA duplex in HEK293T cells by RPA, specifically at their overlapping region. RT‐PCR revealed that this overlapping portion was at least partially protected from RNase degradation (Figure 3H). The combination of lncRNA TBX5‐AS1:2 and *TBX5* mRNA was further validated by RNA‐RNA pull‐down assay (Figure 3I). We then blocked RNA synthesis in HEK293T cells using actinomycin D during a 10h period and measured subsequent levels of *TBX5* mRNA to determine if its stability was augmented by lncRNA TBX5‐AS1:2. The stability of TBX5 mRNA increased in HEK293T cells overexpressing lncRNA TBX5‐AS1:2 compared with NC (Figure 3J).

### 3.8 Hypermethylation of lncRNA TBX5‐AS1:2 in injured heart tissues from TOF patients

**FIGURE 4:**
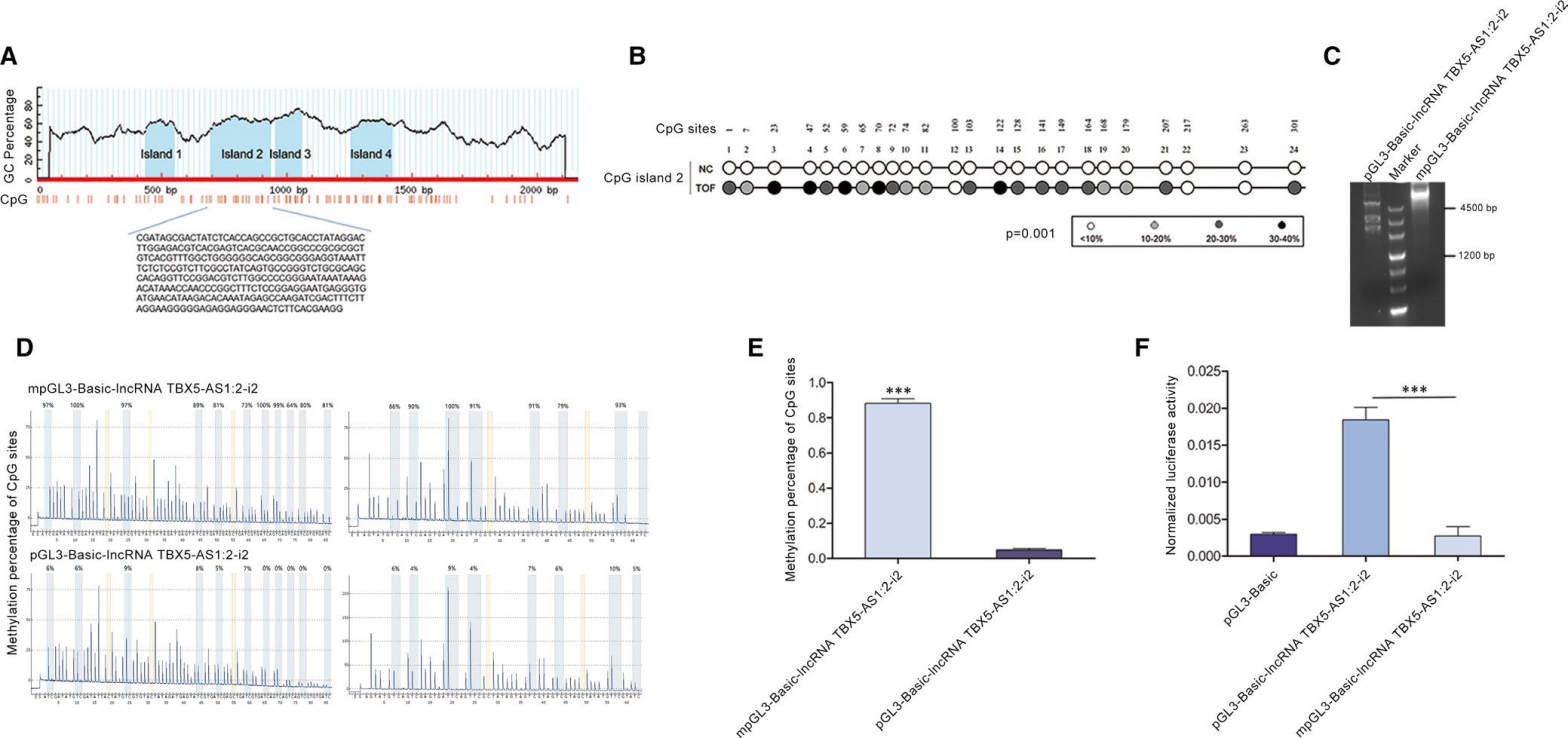
Hypermethylation of CPG island 2 in the promoter caused down‐regulation of lncRNA TBX5‐AS1:2. A, Four CpG islands were predicted in the regulatory sequence of lncRNA TBX5‐AS1:2 by MethPrimer online. B, Every CpG site in lncRNA TBX5‐AS1:2 CpG island 2 was hypermethylated in TOF cardiac tissue samples compared with NC, as detected by BSP for clones. The colour of circles for each CpG site represents methylation percentage. C, The methylated reporter construct pGL3‐Basic‐lncRNA TBX5‐AS1:2‐i2 could not be digested by MSRE whereas the unmethylated construct could be digested. D, The methylation percentage of each CpG site was significantly increased in HEK293T cells transfected with methylated pGL3‐Basic‐lncRNA TBX5‐AS1:2‐i2 according to BSP. E, Overall hypermethylation of CpG island 2 was significant in HEK293T cells transfected with methylated pGL3‐Basic‐lncRNA TBX5‐AS1:2‐i2. (F) Dual‐luciferase reporter assay showed that hypermethylation of CpG island 2 decreased the transcriptional activity of lncRNA TBX5‐AS1:2 in HEK293T cells. Values are mean ± SEM, n = 3, ***P < .0001. BSP, bisulphite sequencing PCR; HEK293T, human embryonic kidney 293; lncRNAs, long non‐coding RNAs; NC, negative control; TOF, Tetralogy of Fallot

DNA methylation is an important factor regulating the expression of lncRNAs. We further explored the mechanism by which lncRNA TBX5‐AS1:2 was down‐regulated in injured heart tissues from TOF patients by predicting the distribution of CpG islands in the lncRNA TBX5‐AS1:2 regulatory sequence using MethPrimer online. Four CpG islands were identified (Figure 4A), of which islands 2 and 3 located in the basal core promoter were selected for investigation of their methylation status in human heart samples. BSP for clones revealed that CpG island 2 was remarkably hypermethylated in the injured heart tissues of TOF patients compared with normal heart tissue (*P* = .001) (Figure 4B and Table S3), but there was no significant difference in CpG island 3 (Table S4).

### 3.9 Hypermethylation of lncRNA TBX5‐AS1:2 caused its down‐regulation

Hypermethylation of CpG islands can inhibit the transcriptional activity of lncRNAs. We therefore transfected methylated and unmethylated lncRNA TBX5‐AS1:2 reporter plasmids containing CpG island 2 into HEK293T cells. The unmethylated construct was digested by MRSEs whereas the methylated construct was not (Figure 4C). BSP revealed that the methylation efficiency of CpG island 2 was increased in HEK293T cells when the construct was methylated (Figure 4D,E). Dual‐luciferase reporter assay revealed that the methylated construct decreased the transcriptional activity of lncRNA TBX5‐AS1:2 in HEK293T cells compared with the unmethylated construct (Figure 4F).

## 4 DISCUSSION

CHD is resulted from abnormal cardiogenesis during early embryonic development. Cardiogenesis involves the orderly proliferation, apoptosis, migration and differentiation of a variety of cells, requiring tight and precise regulation by appropriate spatiotemporal gene expression.^26^ Epigenetic modifications, including lncRNAs, have recently been found to play an important role in this process. What's more, lncRNA is the research hotspot in epigenetics. LncRNAs are classified into five major subclasses according to their relative gene location: antisense, intergenic, overlapping, intronic and full lapping.^27^ All types of lncRNA can regulate their target molecules at the pre‐transcriptional, transcriptional or post‐transcriptional level by binding to DNA, RNA or protein.^28^, ^29^ Previous studies indicated that lncRNAs could affect the cellular process of cardiogenesis by regulating gene expression through a variety of mechanisms,^30^ but most of these studies involved lncRNAs identified in mouse models and/or studied in mouse cell lines.^31^, ^32^, ^33^ However, lncRNAs are not highly conserved between humans and mice,^34^, ^35^ and a better understanding of the lncRNA expression profile in human heart, especially foetal heart, is of crucial importance. TOF is a typical complex CHD, and aberrantly expressed lncRNAs and their actions and gene regulation mechanisms in TOF are therefore likely to be representative. We therefore searched the GEO repository for lncRNA expression profile data for the human heart and mined RNA‐Seq data for foetal and adult heart tissues. We identified 277 lncRNAs and 47 mRNAs related to heart development or CHD that were differentially expressed between foetal and adult hearts by bioinformatics analysis. The regulation of some lncRNAs on several mRNAs may be involved in heart development.

Among the differentially expressed lncRNAs, we focused on the novel antisense lncRNA TBX5‐AS1:2, which showed a relatively high fold change in foetal compared with adult heart, and predicted to target its cognate coding mRNA *TBX5.* Antisense lncRNAs account for approximately 50%‐70% of all lncRNAs and are transcribed from the opposite strand of their endogenous sense counterparts.^36^ An important functional characteristic of antisense lncRNAs is their ability to regulate the expression of their endogenous genes.^37^ Both lncRNA TBX5‐AS1 and *TBX5* were down‐regulated in injured heart tissue from TOF patients. We therefore performed in vitro experiments to clarify the molecular mechanism underlying this clinical phenomenon. Given that no human myocardial cell lines are available and lncRNA TBX5‐AS1:2 has no homologous sequence in mice, we used the tool cell line HEK293T, as in previous studies of CHD or human heart development.^38^, ^39^, ^40^ The function of lncRNAs is determined by their subcellular localization. Nucleoplasmic separation experiments and RNA‐FISH assay showed that lncRNA TBX5‐AS1:2 was mainly located in the nucleus. Antisense lncRNAs in the nucleus have been found to act at nearly every level of gene regulation, based on molecular interactions.^41^, ^42^ Among these, accumulating evidence supports the importance of post‐transcriptional RNA‐RNA interactions.^43^, ^44^, ^45^, ^46^ Sense RNA and antisense lncRNA transcripts form RNA‐RNA duplexes by virtue of their ability to base pair. Consequently, antisense lncRNAs can act as highly specific sensors of mRNA, with this interaction resulting in different post‐transcriptional outcome, including altered mRNA stability.^47^, ^48^ We observed that targeting the non‐overlapping regions of lncRNA TBX5‐AS1:2 with selective shRNAs reduced *TBX5* mRNA and protein abundances in vitro. LncRNA TBX5‐AS1:2 and *TBX5* formed an RNA duplex by RNA‐RNA interaction, thus preventing *TBX5* mRNA degradation and increasing the mRNA stability of *TBX5*. In addition, lncRNA TBX5‐AS1:2 knock‐down significantly inhibited HEK293T cell proliferation, which could be rescued by overexpression of *TBX5*. These results showed that the influence of lncRNA TBX5‐AS1:2 on cell proliferation may be mediated by its interaction with *TBX5*. *TBX5* is a member of the T‐box transcription factor family primarily known for its role in cardiac development.^49^, ^50^, ^51^, ^52^ Mutations or abnormal expression of *TBX5* can increase the risk of CHD, including TOF.^12^, ^53^ Furthermore, non‐coding transcripts may influence heart development by targeting *TBX5*.^54^, ^55^ As a complex developmental process, cardiogenesis includes cell proliferation, which contributes to cardiac growth and regeneration,^56^, ^57^ and decreased cell proliferation comprises part of the clinical phenotype of TOF.^58^, ^59^ Moreover, *TBX5* plays a critical regulatory role in cell proliferation during cardiogenesis.^60^, ^61^ As an epigenetic mechanism, DNA methylation may play important roles in gene expression and regulation. Furthermore, abnormal promoter methylation of lncRNAs was shown to be connected to their dysregulation yy.^62^, ^63^ Hypermethylation of the lncRNA TBX5‐AS1:2 promoter was accordingly detected in injured heart tissue from patients with TOF, associated with lncRNA TBX5‐AS1:2 down‐regulation in vitro.

We therefore suggest that the previously unknown non‐coding antisense transcript lncRNA TBX5‐AS1:2 may be involved in TOF. Hypermethylation could mediate the down‐regulation of lncRNA TBX5‐AS1:2, leading to decreased *TBX5* mRNA and protein expression via an RNA‐RNA interaction regulatory mechanism to inhibit cell proliferation in TOF. However, further studies are needed to determine the detailed mechanisms by which lncRNA TBX5‐AS1:2 modulates cardiac development and its function. Moreover, if there are ideal human cardiomyocyte lines or cardiac stem cells and the parallel data can be obtained from in vitro experiments with these cell lines, the conclusions will be more credible. In summary, this study identified the novel lncRNA TBX5‐AS1:2 as being down‐regulated in human TOF heart tissue and further explored its part of function in cardiac development by in vitro experiments.

## CONFLICT OF INTERESTS

The authors declare no conflict of interest.

## AUTHOR CONTRIBUTIONS

JM, DM and GH were responsible for the idea, project design and concept of the paper. JM performed bioinformatics analysis. WS, WC and XM collected the clinical samples and information. SC, JM, LH and BZ performed the clinical sample detection and in vitro experiments. JM, SC, DM and GH wrote, edited and revised the manuscript. All authors read and approved the manuscript.

## Supporting information

Table S1

Table S2

Table S3

Table S4

## Data Availability

The data sets used and/or analysed during the current study are available from the corresponding author on reasonable request.
