# Supplementary material for: Hypermethylation‐mediated down‐regulation of lncRNA TBX5‐AS1:2 in Tetralogy of Fallot inhibits cell proliferation by reducing TBX5 expression
Source: J Cell Mol Med. 2020 May 5;24(11):6472–84. doi: 10.1111/jcmm.15298 (PMC7294119; doi:10.1111/jcmm.15298)
Supplement: Supplementary file 3 — Table S3 [file JCMM-24-6472-s003.docx]

**Table. S3 The methylation rate of every CpG sites in lncRNA TBX5-AS1:2-island 2 in heart tissues of NC and TOF**

| CpG  group | 1 | 7 | 23 | 47 | 52 | 59 | 65 | 70 | 72 | 74 | 82 | 100 | 103 | 122 | 128 | 141 | 149 | 164 | 168 | 179 | 207 | 217 | 263 | 301 | Total |
| --- | --- | --- | --- | --- | --- | --- | --- | --- | --- | --- | --- | --- | --- | --- | --- | --- | --- | --- | --- | --- | --- | --- | --- | --- | --- |
| NC | 0.05 | 0.00 | 0.03 | 0.00 | 0.03 | 0.08 | 0.03 | 0.05 | 0.00 | 0.00 | 0.00 | 0.00 | 0.00 | 0.00 | 0.05 | 0.00 | 0.03 | 0.05 | 0.05 | 0.05 | 0.08 | 0.08 | 0.03 | 0.03 | 0.03 |
| TOF | 0.24 | 0.16 | 0.32 | 0.34 | 0.26 | 0.32 | 0.18 | 0.34 | 0.28 | 0.16 | 0.12 | 0.08 | 0.2 | 0.3 | 0.22 | 0.24 | 0.24 | 0.24 | 0.12 | 0.1 | 0.22 | 0.08 | 0.08 | 0.22 | 0.211 |
| *t* value | 3.657 | 5.761 | 4.939 | 7.496 | 3.287 | 3.517 | 3.245 | 5.581 | 12.35 | 3.528 | 2.828 | 1.210 | 3.220 | 5.916 | 2.784 | 8.641 | 3.391 | 3.010 | 2.058 | 0.882 | 2.081 | 0.158 | 1.151 | 4.082 | 6.121 |
| *p* value | 0.008 | 0.001 | 0.002 | 0.000 | 0.013 | 0.010 | 0.014 | 0.001 | 0.000 | 0.010 | 0.026 | 0.266 | 0.015 | 0.001 | 0.027 | 0.000 | 0.012 | 0.020 | 0.079 | 0.407 | 0.076 | 0.879 | 0.287 | 0.005 | 0.001 |

NC, normal control; TOF, Tetralogy of Fallot
