# Supplementary material for: Hypermethylation‐mediated down‐regulation of lncRNA TBX5‐AS1:2 in Tetralogy of Fallot inhibits cell proliferation by reducing TBX5 expression
Source: J Cell Mol Med. 2020 May 5;24(11):6472–84. doi: 10.1111/jcmm.15298 (PMC7294119; doi:10.1111/jcmm.15298)
Supplement: Supplementary file 4 — Table S4 [file JCMM-24-6472-s004.docx]

**Table. S4 The methylation rate of every CpG sites in lncRNA TBX5-AS1:2-island 3 in heart tissues of NC and TOF**

| CpG  group | 1 | 6 | 9 | 35 | 87 | 100 | 119 | 133 | 160 | 175 | 184 | 191 | 264 | 267 | 274 | total |
| --- | --- | --- | --- | --- | --- | --- | --- | --- | --- | --- | --- | --- | --- | --- | --- | --- |
| NC | 0.10 | 0.18 | 0.18 | 0.15 | 0.18 | 0.13 | 0.16 | 0.13 | 0.13 | 0.05 | 0.10 | 0.08 | 0.13 | 0.10 | 0.03 | 0.12 |
| TOF | 0.14 | 0.21 | 0.16 | 0.21 | 0.12 | 0.10 | 0.14 | 0.14 | 0.12 | 0.05 | 0.15 | 0.14 | 0.09 | 0.10 | 0.07 | 0.13 |
| *t* value | 0.445 | 0.314 | 0.227 | 0.429 | 0.828 | 0.346 | 0.270 | 0.178 | 0.072 | 0.034 | 0.589 | 1.022 | 1.480 | 0.055 | 1.292 | 0.195 |
| *p* value | 0.668 | 0.761 | 0.827 | 0.679 | 0.432 | 0.738 | 0.794 | 0.863 | 0.945 | 0.974 | 0.572 | 0.337 | 0.178 | 0.958 | 0.232 | 0.850 |

NC, normal control; TOF, Tetralogy of Fallot
